# Risk Analysis between Hip Strength with Hamstring Injuries among Professional Youth Footballers in a Single Malaysian Football Club

**DOI:** 10.5704/MOJ.2511.002

**Published:** 2025-11

**Authors:** M Azwan-Aziz, MY Yunus, AH Ahmad-Shushami

**Affiliations:** 1 Department of Orthopaedics and Traumatology, Universiti Kebangsaan Malaysia, Kuala Lumpur, Malaysia; 2 Department of Sports Medicine, Universiti Malaya, Kuala Lumpur, Malaysia

**Keywords:** injuries, sports injuries, football, youth, incidence rate

## Abstract

**Introduction:**

There is paucity of research regarding the incidence of hamstring injuries and its inherent causes within youth Malaysian football contexts. We aim to investigate the incidence of hamstring injuries among youth footballers and analyse the risk between intrinsic risk variables (anthropometric and hip strength) and the risk of hamstring strain injuries (HSI).

**Material and Methods:**

This was a prospective cohort study involving 72 youth Malaysian professional footballers from a single prestigious club. This study was conducted during the 2023 Malaysian football league. Pre-season medical evaluations encompassed demographic information, anthropometric measurements, and isometric strength examinations of the hamstrings, quadriceps, hip abductors, and hip adductors. Injury surveillance was conducted during the season.

**Results:**

The incidence of HSI in this study was 0.331 injuries per 1000 H, with incidence of injury during match higher 2.79 injuries per 1000 H compared to training 0.216 injuries per 1000 H. There was no hamstring injuries reported in U20. Forty-one (56.9%) has hamstring to quadriceps (H:Q) ratio <0.6 and forty-six (63.9%) has hip abductor to adductor ratio <0.8. The binary logistic regression analysis revealed increasing age (OR: 1.227, CI: 0.98 – 5.03), increased body mass index (OR: 1.79, CI: 0.415 – 7.77), increased body fat mass (OR: 1.39, CI: 0.33 – 5.89), and low H:Q ratio (OR: 4.274, CI: 0.347 – 58.1), increase the risk of HSI.

**Conclusion:**

Injury prevention programs in youth footballers should incorporate these modifiable risk factors into account to reduce the risk of hamstring injuries.

## Introduction

There is limited research on hamstring strain injury (HSI) among youth Malaysian footballers. Youth is defined as individuals aged 15 – 24 years old^1^. This age group is pivotal as it involves dramatic development in physical, psychological, and social, concurrently taking place^2^. It is essential to devise and execute strategies (e.g., neuromuscular training, appropriate rule enforcement, and a focus on safe play) to prevent and mitigate the incidence and severity of football-related injuries among youth players^3^. Thus, identifying the hamstring incidence and possible risk factors is important to implement preventive strategies. While there are rising number of research focusing on injury prevalence in youth footballers^4^, there are a few limitations identified; (i) none of the studies focuses on hamstring injury alone, (ii) significant discrepancies in the reporting of hamstring injuries between studies hinder appropriate analysis, (iii) none of the research utilise a Malaysian population. Injury prevention is a crucial factor facilitating the exponential growth and performance of young athletes. Identifying the risk factors of HSI in youth Malaysian footballers is essential for the advancing growth and potential in the Malaysian football landscape.

Football skills involve sprinting, acceleration and deceleration, rapid change in direction, and jumping. Such skills predispose footballers to an increased risk of developing hamstring injury^5,6^. During high-speed running, the hamstring muscle plays a major role by contracting eccentrically to slow down knee extension during the terminal swing phase^7^. On the other hand, hip abductor and adductor are important stabilisers of the lumbopelvic during high-speed running^8^. Thus, it is important to look at overall hip function, rather than focusing on single muscle group when identifying risk factors of HSI. Most of hamstring research focuses on hamstring and quadriceps strength. A meta-analysis did not support hamstring peak torque as a risk factor for hamstring muscle strain-type injuries (SDM= −0.24, 95% CI −0.85 to 0.37, p=0.44, I2=58%), while supporting an increase in quadriceps peak torque as a risk factor for hamstring muscle strain-type injuries (SDM= 0.43, 95% CI 0.05 to 0.81, p=0.03, I2=0)9. This study also demonstrated no difference between hamstring to quadriceps (H:Q) strength ratio <0.6 and >0.6 (SDM= −0.50, 95% CI −1.17 to 0.18, p=0.15, I2=70%)^9^. However, it utilised isokinetic using concentric and eccentric strength measurement, instead of isometric. Most importantly, none of the studies focuses on hip abductor and adductor strength, even though this muscle group plays a pivotal role in stabilising the hip^9^. High-speed running is strongly associated with an increase incidence of hamstring muscle strain injuries in sports, especially during running and kicking motion^10^. In a study spanning two playing seasons of Australian football, Verrall *et al* documented that a substantial majority (65 out of 69) of confirmed hamstring muscle strain injuries were sustained during running activities^11^. Brooks *et al* reported that approximately 10% of hamstring muscle strain were associated with kicking activities^12^. Furthermore, their findings indicated that these kicking-related hamstring injuries resulted in greater time lost from play compared to injuries sustained during other activities. Stretching type of injury (kicking) such as kicking results longer return to play as compared to eccentric type (running)^13^.

To our knowledge, this would be the first study in Malaysia that investigates the incidence of hamstring injury in youth Malaysian Professional footballers and assesses the risk of HSI in hip strength deficit (hip abductor, adductor, hamstring, and quadriceps strength) and lower limb strength imbalance (hamstring to quadriceps ratio and abductor to adductor ratio). Our main objectives are (i) to investigate the incidence of HSI in youth Malaysian Professional footballers and (ii) to investigate the risk of HSI in hip strength deficit (hip abductor, adductor, hamstring, and quadriceps strength) and lower limb strength imbalance (hamstring to quadriceps strength ratio and abductor to adductor strength ratio) in youth Malaysian professional footballers.

## Materials and Methods

This was a prospective cohort study, on 72 youth Malaysian professional footballers, aged 15–24 years old, from a single renowned football club. However, the minimum age of footballers in this club were 18 and above. All Malaysian footballers were included in this study. Age >24 years old and non-Malaysian footballers were excluded from this study.

The pre-season assessments were done at the football club itself. Footballers were asked to rest for one day before medical assessments. Using a standardised registry, demographic data were collected: age, gender, position played, mechanism of injury, previous history of hamstring injury, and dominant leg. Name, identification number, and football club name were collected but were excluded from the analysis. Written consent has been taken prior to the study with the footballers.

Anthropometric measurements were taken by a sports medicine trainee using Inbody 370 Body Composition Analysis. Footballers were asked to fast 6 hours prior to the test. Height, weight, body mass index (BMI), body fat mass, and skeletal muscle mass were recorded in the registry by examiner 1.

Assessment of muscle strength was preceded by a 15-minute warm-up. Adequate familiarisation to each testing device and measurement was given with each subject. Hamstring, quadriceps, hip adductor, and hip abductor isometric torque assessments were carried out by other examiner (examiner 2) using JTECH Commander Powertrack Muscle Dynamometer manual muscle testing. The techniques of the manual muscle testing are outlined in Table I. Three measurements were obtained, and maximum values were chosen. We chose objective muscle strength testing using a handheld dynamometer as it has a (i) high correlation between hip, knee and ankle strength compared to isokinetic, (ii) cheaper and (iii) is applicable as bedside examination in clinic and office^14^.

**Table I:** Assessments of isometric lower limb strength using handheld dynamometer.

	Position of footballers	Placement of dynamometer	Testing
Hamstring	Sitting with knee flexion 90°	5cm from tibial tuberosity	Flex the knee against resistance
Quadriceps	Sitting with knee flexion 90°	5cm from imaginary line formed from lateral and medial femoral condyle	extend the knee against resistance
Hip abductor	Sitting with knee flexion 90°	At the lateral femoral condyle	Abduct the hip against resistance
Hip adductor	Sitting with knee flexion 90°	At the medial femoral condyle	Adduct the hip against resistance

The data collection was conducted based on the method proposed by the Injury Consensus Group, which was established under the Federation Internationale de Football Association Medical Assessment and Research Centre (F-MARC)^15^. All injuries during training and competition reported by the footballers to either team physician and/or team physiotherapist, will be recorded, throughout this season in google form. HSI was defined as any injury occurred over the posterior thigh, diagnosed as muscle strain, that results in time loss^16^. Athlete’s name, identification number, date of injury, injury that occurred during training or competition, site of injury, and time loss, were included in this study. The severity of the injury was further classified as minimal (1–3 days), mild (4–7 days), moderate (8–28 days), and severe (>28 days), according to the number of days of absence from training and matches^16^.

Hamstring to Quadriceps ratio (H:Q) is an independent risk factor for HSI and optimum value of the conventional H:Q should be 0.6017. Thus, 0.6 value was chosen as the cutoff point for low H:Q ratio. Adductor to Abductor strength ratio (Add:Abd) has never been explored as an independent risk factor for hamstring injury. Hip Add:Abd strength ratio close to 0.8 was observed in asymptomatic footballers^18^. Tyler *et al* (2001) hip adductor to abductor strength ratio was the best predictor of a future adductor strain. Relative risk for an adductor strain was 17:1 based on a hip Add:Abd ratio less than 80%^19^. Thus, cutoff point of 0.8 was chosen as low Add:Abd ratio. Interlimb deficit is defined as the differences between two limbs muscle groups.

A total of 72 footballers, consisted of 3 teams; senior team, under 23 (U23) and under 20 (U20). Descriptive analysis was applied, demographic data (age), anthropometric data (weight, height, body mass index, body fat mass, skeletal muscle mass), baseline hamstring and quadriceps strength, and interlimb deficit were expressed as mean and standard deviation. The differences in mean of hamstring, quadriceps, hip abductor, and hip adductor strength between dominant and non-dominant leg were compared using the student t test and p-value <0.05 was considered significant. The injury incidence was reported as the number of injuries per 1000 player hours, including match and training injury incidence. The risk analysis was performed between the lower limb strength, deficit, previous HSI, and anthropometry and the rate of HSI was examined by backward stepwise binary logistic regression (Wald, inclusion probability p≤0.10) with OR analysis for estimating the simultaneous effects of several predictors instead of relative risk estimates^20,21^. Few independent variables have been included in the regression analysis such as age, anthropometric assessments (weight, body mass index, skeletal muscle mass, body fat mass), and hip strength (interlimb hamstring deficit, interlimb quadriceps deficit, interlimb hip abductor deficit, interlimb hip adductor deficit, hamstring to quadriceps strength ratio, hip adductor to abductor strength ratio). Body fat percentage, and maximum hip strength (hamstring, quadriceps, hip abductor, hip adductor) were removed as it has high collinearity with variance inflation factor >10. This study was approved by Medical Research Ethic Committee, University Malaya Medical Centre (MREC:202216-11002).

## Results

A total of 72 footballers were included in this study; 24 of them from senior team, 22 of them from under 23 team, and 26 of them were from under 20 team. The mean age of the footballers was 20.5 years ± 1.6 years, ranging from 18 – 24 years old. All footballers were male (100%). Seven (9.7%) were goalkeepers, 20 (27.8%) were defendants, 23 (31.9%) were midfielder and 22 (30.6%) were strikers. Sixty-three (87.5%) of the footballers have no history of HSI previously. Baseline data were outlined in Table II.

**Table II:** Baseline characteristics between senior team, Under 23 and Under 21.

		Total (n =72)	Senior (n =24)	Under 23 (n =22)	Under 20 (n =26)
Age (years) Anthropometric					
	Height				
	Weight (kg)				
	BMI (kg/m^2^)				
	Body Fat Mass (kg)				
	Skeletal muscle mass (kg)				
Position					
	Goalkeeper	7 (9.7)☆	2 (8.3)☆	1 (4.5)☆	4 (15.4)☆
	Defender	20 (27.8)☆	5 (20.8)☆	7 (31.8)☆	8 (30.8)☆
	Midfielder	23 (31.9)☆	9 (37.5)☆	8 (36.4)☆	6 (23.1)☆
	Striker	22 (30.6)☆	8 (30.8)☆	6 (27.3)☆	8 (30.8)☆
Dominant Leg					
	Right	51 (70.8)☆	17 (70.8)☆	15 (68)☆	19 (73)☆
	Left	21 (29.2)☆	7 (29.2)☆	7 (32)☆	7 (27)☆
Previous hamstring Injury					
	No	63 (87.5)☆	22 (91.7)☆	17 (77.3)☆	24 (92.3)☆
	Yes	9 (12.5)☆	2 (8.3)☆	2 (22.7)☆	2 (7.7)☆

* expressed as mean ± SD ☆ expressed as frequency (percentage)

The mean preseason strength of hamstring, quadriceps, and hip abductor is higher in dominant leg compared to non-dominant leg (Table III) and the mean preseason strength of hip adductor is higher in non-dominant leg compared to dominant leg. Twenty (27.8%) reported hamstring interlimb strength deficit >15%, 16 (22.2%) reported quadriceps intralimb strength deficit >15%, 20 (27.8%) reported hip abductor interlimb strength deficit >15%, 12 (16.7%) reported hip adductor intralimb strength deficit >15%. Interestingly, 41 (56.9%) have H:Q ratio of less than 0.6, and 46 (63.9%) has hip Add:Abd ratio <0.8. Baseline muscle assessments are outlined in Table IV.

**Table III:** Comparison interlimb lower limb hamstring and quadriceps strength.

	Dominant		Non-dominant		P-value
	Mean (SD)	CI	Mean (SD)	SD	
Maximum isometric hamstring strength	37.5 (7.02)	35.9 – 39.2	36.27 (7.8)	34.4 – 38.1	<0.005
Maximum isometric quadriceps strength	72.9 (14.6)	69.5 – 76.4	72.1 (14.9)	68.6 – 75.6	<0.005
Maximum isometric hip abductor strength	42.6 (7.19)	40.5 – 44.7	42.1 (8.93)	40.2 – 43.9	<0.005
Maximum isometric hip adductor strength	40.7 (9.39)	38.5 – 42.9	42.1 (9.91)	39.6 – 44.3	<0.005

Notes - Comparison Interlimb lower limb hamstring and quadriceps strength using student t-test and p-value <0.005 is considered significant.

**Table IV:** Intralimb and interlimb deficits in 72 footballers.

	Group	N	%	Mean deficit	SD
Hamstring intralimb strength deficit	<15%	52	72.2	11.76	8.9*
	>15%	20	27.8		
Quadriceps intralimb strength deficit	<15%	56	77.8	10.2	7.38*
	>15%	16	22.2		
Hamstring to Quadricep strength ratio	<0.6	41	56.9	0.53	0.114*
	>0.6	31	43.1		
Hip abductor intralimb strength deficit	<15%	52	72.2	9.29	6.97*
	>15%	20	27.8		
Hip adductor intralimb strength deficit	<15%	60	83.3	8.6	6.3*
	>15%	12	16.7		
Hip Abductor to adductor strength ratio	<0.8	13	18.1	0.96	0.17*
	>0.8	59	81.9		

Notes – Intra limb muscle strength deficit was divided into two groups; <15% and >15%, and mean deficit percentage were calculated. Hamstring quadriceps ratio was divided into two groups; <0.6 and >0.6 and mean deficit percentage were calculated. Hip Abductor to adductor strength ratio divided into two groups; <0.8 and >0.8 and mean deficit percentage were calculated.

Throughout season, there were a total of 76 injuries reported. HSI was the highest (n=11 injuries, 8 footballers, 14.4%), followed by adductor strain (n=14, 16.27%), and ankle sprain (n=7, 8.1%). One footballer suffered three times recurrent HSI throughout the study with recurrence rate of 9%. All hamstring injuries were caused by non-contact injuries; 10 (90.9%) were due to sprinting, and 1 (9.1%) due to kicking. There were no hamstring injuries reported in U20. The incidence of HSI in this study was 0.331 injuries per 1000 H, with incidence of injury during match higher 2.79 injuries per 1000 H compared to training 0.216 injuries per 1000 H, as per Table V. Senior team reported higher incidence of injuries compared to U23 (0.734 vs 0.276 injuries per 1000 H). One (12.5%) has 1–3 days’ time loss, 5 (62.5%) has 8-28 days’ time loss, and 2 (25%) have time loss >28 days.

**Table V:** Incidence of hamstring strain injuries among 72 footballers.

	Total Injuries (n)	Hamstring injury(n)			Exposure (h)			Hamstring injury incidence (injury/1000h)		
Team		Total	Match	Training	Total	Match	Training	Total	Match	Training
Senior	61	6	2	4	8170.5	511.5	7659	0.734	3.91	0.522
Under 23	9	2	1	1	7227.5	313.5	6914	0.276	3.18	0.144
Under 20	6	0	0	0	8749.5	247.5	8502	0	0	0
Total	76	8	3	5	24147.5	1072.5	23075	0.331	2.79	0.216

The binary logistic regression analysis revealed that several factors were significantly associated with hamstring strain injuries. Increasing age significantly increased the risk of HSI (OR: 1.227, 95% CI: 0.98 – 5.03, p<0.05). For anthropometric assessment, increased body mass index (OR: 1.79, 95% CI: 0.415 – 7.77), increased skeletal muscle mass (OR: 1.39, 95% CI: 0.173 – 11.2), and increased body fat mass (OR: 1.39, 95% CI: 0.33 – 5.89) were positively associated with injury risk. Additionally, a no history of previous hamstring injury reduces risk of hamstring strain injury (OR: 0.085, 95% CI: 0.007-1.038, p<0.05).

Regarding muscle strength, low hamstring to quadriceps strength ratio increased risk of HSI (OR: 4.274, 95% CI: 0.347 – 58.1). On the other hand, interlimb hamstring deficit <15 % and hip Add:Abd ratio <0.8 decreased the risk of HSI (OR: 0.953, 95% CI: 0.114 – 8) and (OR: 0.425, 95% CI: 0.046 – 3.95) This is outlined in Table VI. This trend was not significant at the 0.05 level, probably owing to the small sample size and small number of footballers having this injury.

**Table VI:** Binary Regression analysis on predictors of HSI among 72 footballers.

Variables		B	Binary OR	regression P-value	95% CI
Age		0.801	1.227	0.05	0.98 – 5.03
No Previous history of hamstring injury		-2.461	0.085	0.05	0.007-1.038
Anthropometric	Weight (kg)	-0.344	0.709	0.591	0.2 – 2.51
	Body mass index (kg/m2)	0.585	1.79	0.433	0.415 – 7.77
	Body fat mass (kg)	0.33	1.39	0.651	0.33 – 5.89
	Skeletal muscle mass (kg)	0.33	1.39	0.893	0.173 – 11.2
Strength	intra-limb hamstring asymmetry <15%	-0.048	0.953	0.965	0.114 – 8
	intra-limb Quadriceps asymmetry <15%	2.67	14.4	0.931	0.09 – 2169.3
	Hamstring: Quadriceps <0.6	1.453	4.274	0.275	0.347 – 58.1
	intra-limb hip abductor asymmetry <15%	-0.088	0.916	0.938	0.099 – 8.46
	intra-limb hip adductor asymmetry <15%	- 0.913	0.401	0.569	0.017 -9.29
	Adductor: abductor ratio <0.8	-0.856	0.425	0.452	0.046 – 3.95

## Discussion

A total of 72 professional footballers were examined pre-season and followed-up for a total of 10 months, throughout the season. The incidence of hamstring injuries in our study was 0.331 in 1000 exposure hours. In a systematic review looking into 3868 adult footballers, the incidence of HSI reported ranged from 0.3 to 1.9 per 1000 exposure hours^22^. However, none of the studies are in Asian population. This study comprises of adult age group, which showed higher incidence of injuries compared to our study. Looking at a similar age group set (youth), a systematic review of epidemiology of sports injuries in youth footballers reported incidence injuries of 1.21 in 1000 exposure hours around thigh region^4^. None of the studies mentioned hamstring injuries, complicating the conclusion that this prevalence is only attributable to hamstring injuries. Our study reported no injuries from U20. An epidemiological study of 431 young Spanish footballers from under 9 to under 23 age group, found that higher incidence of injuries was seen in older age group (U23)^23^. No hamstring injury was reported in aged under 9 and under 13. Our study reported high incidence of HSI in senior team and U23. Research has shown that hamstring injuries peak at 16 – 25 years old (youth age group)^24^. We postulated three hypotheses of no injuries in U20 group. The higher volume and intensity of the football training and matches in senior team and U23 compared to U20, increases the risk of HSI among senior team^25^. Crowded player calendar in senior team is another risk factor, for increased risk of hamstring injuries^25^. Senior team often had inadequate recovery time for the footballers in between training with a higher number of up to two competitive league matches per week. However, with adequate monitoring and injury prevention programs, we were able to keep the incidence of injury at low number.

Age as non-modifiable risk factor of HSI is controversial and lacks of basic science. Increasing age has 22.7% higher odds of HSI in our study. A meta-analysis supported our findings with seven studies (3199 participants) showing age is a significant risk factor of HSI (SDM=2.5, 95% CI 0.78 to 4.15, p=0.004, I2=99%)^9^. This study also demonstrates increasing age increase risk of HSI by 2.46 times^9^. In Woods *et al* study, youth footballers aged 17 to 22 experienced fewer hamstring strains than older players^26^. Orchard *et al* reported footballers age >23 years old have increased risk of HSI compared to <23 years old^27^. According to Henderson *et al*, for every 1-year increase in age, the odds for sustaining hamstring injury increased 1.78 times in English footballers, lower compared to our study^28^. According to Gabbe *et al*, increasing body mass index and reduced hip flexor flexibility were identified as age-related changes identified, poses increased risk of HSI^29^.

Our study hypothesises that decrease hamstring: quadriceps strength ratio and increase hamstring deficit, will increase the risk of hamstring injury. In our study, footballers with low hamstring: quadriceps ratio have an increased risk of developing acute HSI, while no previous history of hamstring injury, hamstring deficit less than 15% has reduced risk of acute hamstring injury. Muscle strength imbalance surrounding the knee joint has long been recognised as a risk factor for hamstring strain injuries (HSI) and is commonly assessed using the hamstring-to-quadriceps (H:Q) ratio^30^. A low H:Q ratio indicates that the strength capability of the hamstrings is insufficient to counteract the forceful activities of the quadriceps, potentially compromising joint and/or muscle integrity. Similar findings were reported by Lee *et al* on 146 footballers with lower hamstring-to-quadriceps strength ratio concentric H:Q ratio below 50.5% (OR=3.14; 95% CI, 1.37 – 2.22), and a previous injury of HSI (OR=3.57; 95% CI, 3.13 – 8.62) were linked to an increased risk of acute HSI^31^. Croisier *et al* suggested that footballers with H:Q ratio less than 0.55 had 4.66 folds increased risk of HSI^21^. The normal H:Q ratio is considered to be 50% to 80% as averaged through the full range of knee motion, with a higher ratio at faster speeds. Whilst meta-analysis reported no significant difference between low and normal low H:Q ratio, all of the studies used isokinetic machine that examined isotonic strengths, at various velocities, which reproduced different injury risks^9^. There were no studies in the meta-analysis utilise handheld dynamometer, which is cost-effective, and can be used in office setting or at pitch side. There were studies that examined the H:Q ratio isometrically, had comparable results to our study^32,33^. Thus, office handheld dynamometer, can potentially be recommended to assess H:Q pre-season, to predict future HSI, and prevention intervention can be applied to reduce the risk.

Previous hamstring injury is a well-described risk factor for future HSI in the literature^9^. Similar findings were reported in our study. One footballer in our study, who suffered from recurrent hamstring injury throughout this season, had previous hamstring injury. Bennell *et al* reported that previous history of HSI in footballers increased the risk of future HSI 2.1 times more^34^. Koulouris *et al* noticed an increasing trend of recurrent HSI in previous history of HSI (p=0.07)^35^. Although previous injury is seen as a non-modifiable risk factor, it has been highlighted that previous injury led to reduced eccentric hamstring strength, interlimb asymmetries, and short biceps femoris fascicle length^36,37^. It could be hypothesised that improving these physical attributes may reduce the risk of recurrent HSI^36,37^. Therefore, the key to reducing future injury risk in these players is to identify reversible risk factors associated with this group, or deficits following injury.

Looking at a new perspective, hip adductor to abductor ratio is a potential risk factor for HSI. In our study, adductor: abductor ratio <0.6 has reduced risk of HSI. This indicates that hip abductor is stronger compared to hip adductor. A kinematic analysis reported that anterior trunk sway and contralateral pelvic drop while standing on one leg increased the load on the hamstrings^38^. This suggests that an imbalance in the hip abductors and adductors can result in pelvic instability, hence increasing the risk of hamstring injury. This study shows the importance of having good hip abductor to maintain hip stability and reduce risk of injuries.

Hamstring injury though has been extensively at international level, we have limited studies on incidence of HSI and possible internal risk factors in Malaysian football settings. This study is a stepping stone research, for more football injury prevention studies among Malaysian football population. Our study can serve as a commendable starting point, examining HSI incidence and risk factors among professional footballers in Southeast Asia, particularly Malaysia, which possesses distinct population characteristics and racial compositions compared to European professional footballers. This study outlined that assessments of HSI risk factors could be done in office, without having to use sophisticated machine. Office handheld dynamometer is a cost-efficient, real-time results, and clinician-friendly, is recommended as part of prevention program assessments for HSI. Nevertheless, there are some limitations in our study. Numerous risk factors such as athlete’s risk factors, environmental risk factors, psychological, and biomechanical risk factors were not accountable in our study. Further exploration of these factors would be advantageous in Malaysian football settings. The sample size is small. However, that reflects the number of footballers available in a football clubs. A longer prospective cohort study would be recommended in the future. Moreover, this does not represent the Malaysian population. There was possibility of under-reported, self-treated minor hamstring strain among U20, as there we hamstring injuries reported in that group. A cut off value of H:Q ratio and hip Add:Abd ratio of 0.6 and 0.8, respectively may not represent Malaysian cohort. Thus, this value should be explored further in future study.

## Conclusion

This study investigated the incidence and risk factors of hamstring injuries among professional footballers in Malaysia. The incidence of HSI is lower compared to the adult European, and Australian population, indicating that injury prevention programs in youth Malaysian footballers are up to par with the international standard. Increasing age, increasing BMI, and body fat mass, previous injury history, and low H:Q ratio <0.6 were identified as significant risk factors for HSI in youth footballers. Thus, recommendation to maintain body mass index, and focus on hamstring strengthening program especially footballers with previous history of hamstring injury should be the main focus of preventive program.
